# Supplementary material for: From sequence to enzyme mechanism using multi-label machine learning
Source: BMC Bioinformatics. 2014 May 19;15:150. doi: 10.1186/1471-2105-15-150 (PMC4229970; doi:10.1186/1471-2105-15-150)
Supplement: Additional file 2 — Java code of ml2db. Additional file ml2db_code.tar.gz contains the Java source code to run the multi-label machine learning experiments and save the results to database. The code’s Javadoc is included. [file 1471-2105-15-150-S2.zip › additional file 2/ml2db/utils/doc/cern/colt/function/DoubleFunction.html]

DoubleFunction


JavaScript is disabled on your browser.


- Overview
- Package
- Class
- Use
- Tree
- Deprecated
- Index
- Help

- Prev Class
- Next Class

- Frames
- No Frames

- All Classes

- Summary:
- Nested |
- Field |
- Constr |
- Method

- Detail:
- Field |
- Constr |
- Method


cern.colt.function

## Interface DoubleFunction

- All Known Implementing Classes:
  :   AbstractContinousDistribution, AbstractDistribution, MersenneTwister, Pareto, PseudoTruncatedPareto, RandomEngine, TruncatedPareto

  ---

    

  ```
  public interface DoubleFunction
  ```

  Interface that represents a function object: a function that takes a single
  argument and returns a single value.

- - ### Method Summary

    Methods

    | Modifier and Type | Method and Description |
    | --- | --- |
    | `double` | `apply(double argument)` Applies a function to an argument. |

- - ### Method Detail


    - #### apply

      ```
      double apply(double argument)
      ```

      Applies a function to an argument.

      Parameters:
      :   `argument` - argument passed to the function.

      Returns:
      :   the result of the function.


- Overview
- Package
- Class
- Use
- Tree
- Deprecated
- Index
- Help

- Prev Class
- Next Class

- Frames
- No Frames

- All Classes

- Summary:
- Nested |
- Field |
- Constr |
- Method

- Detail:
- Field |
- Constr |
- Method
